# Patterns of kidney function and risk assessment in a nationwide laboratory database: the Brazilian CHECK-CKD study

**DOI:** 10.1186/s12882-024-03588-w

**Published:** 2024-06-04

**Authors:** Murilo Guedes, Paulo Telles Dias, Rosângela R. Réa, Viviane Calice-Silva, Marcelo Lopes, Andrea Araujo Brandão, Andrea Carla Bauer, Andreza Almeida Senerchia, Pedro Túlio Monteiro de Castro e Abreu Rocha, Bruno Bezerra Rosa, Cinthia Montenegro Teixeira, Roberto Pecoits-Filho

**Affiliations:** 1 Center for Epidemiological Research (EPICENTER), Curitiba, Brazil; 2 School of Medicine, Pontificia Universidade Catolica do Parana, Curitiba, Brazil; 3 Dasa, São Paulo, SP Brazil; 4 Endocrinology Unit - SEMPR, Federal University of Paraná (UFPR), Curitiba, Brazil; 5 Pro-rim Foundation, Joinville, Brazil; 6 School of Medicine, University of Joinville’s region - UNIVILLE, Joinville, Brazil; 7 Federal University of Bahia Medical School, Salvador, Brazil; 8 School of Medical Sciences, State University of Rio de Janeiro, Rio de Janeiro, RJ Brazil; 9 School of Medicine, Universidade Federal do Rio Grande do Sul, Porto Alegre, Brazil; 10 Deparment of Nephrology, Federal University of RIo de Janeiro, Rio de Janeiro, Brazil; 11 Nephrology, Hospital São Lucas Copacabana, Rio de Janeiro, Brazil; 12 AstraZeneca Brazil, Cotia, São Paulo, Brazil

**Keywords:** Chronic kidney disease, Screening, Monitoring, Estimated glomerular filtration rate, Brazil, Public health

## Abstract

**Background:**

Chronic kidney disease (CKD) is a global health problem with rising prevalence, morbidity, mortality, and associated costs. Early identification and risk stratification are key to preventing progression to kidney failure. However, there is a paucity of data on practice patterns of kidney function assessment to guide the development of improvement strategies, particularly in lower-income countries.

**Methods:**

A retrospective observational analysis was conducted in a nationwide laboratory database in Brazil. We included all adult patients with at least one serum creatinine assessment between June 2018 and May 2021. Our primary objective was to determine the proportion of patients with estimated glomerular filtration rate (eGFR) evaluations accompanied by predicted levels of urinary albumin-to-creatinine ratio (pACR) assessments within 12 months.

**Results:**

Out of 4,5323,332 serum creatinine measurements, 42% lacked pACR measurements within 12 months. Approximately 10.8% of tests suggested CKD, mostly at stage 3a. The proportion of serum creatinine exams paired with pACR assessment varied according to the CKD stage. Internal Medicine, Cardiology, and Obstetrics/Gynecology were the specialties requesting most of the creatinine tests. Nephrology contributed with only 1.1% of serum creatinine requests for testing.

**Conclusion:**

Our findings reveal that a significant proportion of individuals with a creatinine test lack an accompanying urinary albuminuria measurement in Brazil, contrary to the recommendations of the international guidelines. Non-Nephrologists perform most kidney function evaluations, even among patients with presumable advanced CKD. This highlights the urge to incorporate in clinical practice the early detection of CKD and to encourage more collaborative multidisciplinary care to improve CKD management.

**Supplementary Information:**

The online version contains supplementary material available at 10.1186/s12882-024-03588-w.

## Background

Chronic kidney disease (CKD) is a common and often unrelenting condition that disproportionally affects individuals with multiple comorbidities and more limited access to healthcare services than the general population. The prevalence, incidence, and mortality rates of CKD have been rising staggeringly worldwide, and significant country variations reflect pervasive disparities on CKD care, from early disease identification to managing associated complications [1]. The rise in CKD risk factors is expected to strain healthcare systems, particularly in non-high-income countries over the next decades [2].

Unrestrained costs and persistent poor outcomes, consistent worldwide, illustrate the importance of preventing CKD progression into kidney failure. Early identification and risk stratification are the cornerstones of kidney failure prevention since they allow timely optimization of care [3]. However, evidence-based recommendations for CKD screening and monitoring are not implemented uniformly across different healthcare areas and systems [4]. The ensuing uncertainty results in differences in how countries develop and implement guidelines for CKD screening and monitoring, which may impact patient outcomes, particularly with the increasing availability of evidence-based safe and effective CKD-modifying therapies [5, 6].

Disparities in access to care and healthcare system fragmentation, particularly in countries where public and private healthcare systems coexist, complicate the process of CKD screening and management [7]. In countries such as Brazil, the problem begins with inconsistent screening and stratification strategies and extends to poor care transition from primary to secondary networks, resulting in late referrals and inadequate preparation for kidney replacement therapies. The absence of nationwide implementation of a CKD care program exacerbates these issues. To date, no reports on national patterns of kidney function assessment across CKD stages and specialties in Brazil are available. Understanding patterns in CKD screening and risk stratification may guide the development of strategies to optimize this process.

In the CHECK-CKD Study in Brazil, we describe a retrospective observational analysis using a nationwide laboratory database aiming to provide insights into practice patterns that can resemble screening or monitoring programs. In the study we use assume that serum creatinine is used to assess kidney function and structural kidney lesion is assessed by appropriate urine tests. Herein we illustrate the varying approaches of medical specialties to kidney disease assessment, focusing on serum creatinine to measure kidney function and tests that quantify proteins in the urine (converted to predicted urinary to albumin-creatinine ratio (pACR)) to assess kidney damage [8]. Furthermore, we evaluate the adherence of practicing physicians in Brazil to guideline recommendations for kidney function assessment, including the frequency of concomitant pACR and Creatinine measurement and longitudinal creatinine-based eGFR evaluation among populations at risk for CKD.

## Methods

### Study design and database

This is a descriptive, non-interventional, retrospective cohort study using real-world data from Diagnosticos da America (DASA) Datalake. DASA is a nationwide integrated healthcare network in Brazil that currently provides medical care to approximately 10 million individuals with private health insurance coverage in Brazil. The database comprises a range of medical tests and biomarkers, including but not limited to complete blood cell count, basic and comprehensive metabolic panels, lipid panels, liver panels, cultures, imaging, and genetic biomarkers. These results are also linked to electronic medical records, which can be manipulated (i.e., filtered, extracted, and analyzed) to monitor laboratory parameters and, if relevant, link the results to specific diseases, conditions, and ICD codes for patients admitted to hospitals maintained by DASA. The data for this study comes from laboratory tests performed by DASA laboratories. Laboratory tests requested by physicians outside of the DASA healthcare network are included in this sample.

During clinical visits, patients voluntarily sign a privacy agreement, granting consent for generating analyses and studies that contribute to the improvement of DASA´s activities in compliance with its Privacy Policy and the General Data Protection Law in Brazil. This protocol was also submitted to the appreciation of the local Research Ethics Committee. Due to the large number of patients and the retrospective nature of the study, a waiver of informed consent was obtained. This study was designed following the STROBE guidelines.

### Patient population and study variables

All adult patients who had at least one serum creatinine assessment from June 2018 and May 2021 were included in this study. For the present study, the extracted data included age, sex, the patient's residential zip code (if not available, the location of the unit where the test was collected will be used), numeric test results, the laboratory unit where the tests were collected, the date of the examination, and the prescribing physician's specialty. We only included laboratory tests ordered in outpatient settings.

All laboratory tests were performed in DASA laboratories. Serum creatinine determination for the assessment of renal function was performed on serum using the standard colorimetric method (Jaffé reaction). The eGFR was calculated using the Chronic Kidney Disease Epidemiology Collaboration (CKD-EPI) equation without race. For the assessment of albuminuria, a spot urine sample was collected and analyzed using the standard protocol with the Turbiquest Plus Microalbuminuria kit, which detects albumin concentrations ranging from 3 to 500 mg/L. The clinical chemical analyzers used for the determinations of serum creatinine and urinary albuminuria across different sites were Roche Cobas, Beckman Au, Abbott Architect, Siemens Centaur, and Siemens Atellica.

Urinary tests that quantify proteinuria were evaluated within 12 months of the index creatinine assessment. Results from semi-quantitative tests were converted into continuous albuminuria measured in mg/g creatinine using validated methods [8]. Specifically, we applied the following equations to convert urinary protein-creatinine ratio (PCR) (Equation 1) or dipstick results (Equation 2) to pACR [8]:

1 $$pACR=exp\ (5.3920\ +\ 0.3072\times log\ (min(PCR/50,1))\ +\ 1.5793\times log(max(min(PCR/500,1),0.1))\ +\ 1.1266\times log(max(PCR/500,1)))$$

2 $$pACR = exp (2.4738\ +\ 0.7539\times (if trace)\ +\ 1.7243\times (if\ +\ )\ +\ 3.3475\times (if++)\ +\ 4.6399\times (if >++))$$

Where “trace”, “+”, “++” and (“>++”) refer to semi-quantitative results.

All converted results are reported as pACR [8]. Diabetes was defined as a single Hb1c > 6.5% or a serum glucose level above 200 mg/dL within 12 months from the index creatinine measurement.

### Objectives

Our primary objective was to describe the proportion of patients who underwent eGFR evaluation and had at least one pACR measurement within 12 months. Our exploratory objectives were to (i) describe the frequency of medical specialties requesting laboratory tests for CKD diagnosis (reported in absolute counts), (ii) determine the proportion of patients with potential evidence of CKD based on single eGFR and pACR measurements in the sample, and (iii) determine the proportion of patients who underwent a follow-up creatinine measurement after an initial eGFR lower than 60/ mL/min/1.73 m2.

### Statistical analysis

Descriptive statistics were calculated to summarize the demographic and clinical characteristics of the study population. Continuous variables were presented as means and standard deviations (SD) or medians and interquartile ranges (IQR), depending on the distribution of the data. Categorical variables were reported as frequencies and percentages. The cumulative incidence of a new serum creatinine assessment among patients with one serum creatinine measurement was calculated as one minus the survival function, which was estimated by the Kaplan-Meier product-limit method. Analyses were stratified by CKD stage using the first serum creatinine result assessment. All analyzes were performed using the software R version 4.1.2.

## Results

**Fig. 1:**
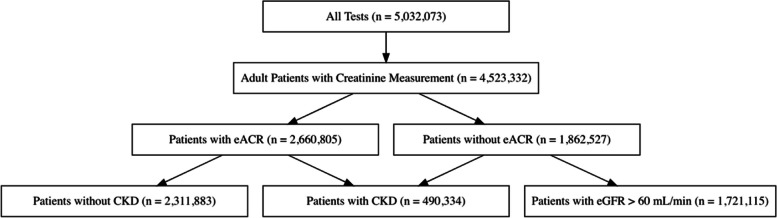
Flow-Chart of Inclusion and Exclusion of Observations in the Sample

Figure 1 depicts the inclusion and exclusion process for the analysis sample. A total of 4,5323,332 serum patients with serum creatinine measurements were included. Approximately 41% of these patients did not have pACR measurements within 12 months, yielding 2,660,805 patients in the sample used for the estimation CKD frequency according to the KDIGO classification. Following the demographic distribution of the Brazilian population, most of the tests were performed in the states of São Paulo (47.6%), followed by Rio de Janeiro (22.6%), and Paraná (9.3%) (Supplementary Figure 1).

Patients had a mean age of 45 years, were mostly female (60%), and up to 10% had diabetes. Overall, 10.8% of tests suggested CKD, mostly at stage 3a (54.1%), followed by 3b (22.1%), G1 (6.8%), G4 (6.1%), G2 (8.3%), and G5 (2.6%). Table 1 describes the CKD stages following the KDIGO classification. The proportion of serum creatinine exams paired with an pACR assessment varied according to CKD stage: G1 (54.9%), G2 (61.2%), G3a (66.7%), G3b (70.7%), G4 (70.2%) e G5 (46.7%). The proportion of laboratory results indicating A3-level albuminuria was 0.3%, 0.4%, 1.2%, 2.5%, 7.3%, and 28.4% from G1 to G5, respectively. Patients with CKD were older (mean age 69 ± 15.2 vs. 47.2 ± 15.4 years) and more likely to have diabetes (22% vs. 8%) than patients without CKD. The geographical distribution was similar between CKD and non-CKD patients.

**Table 1:**
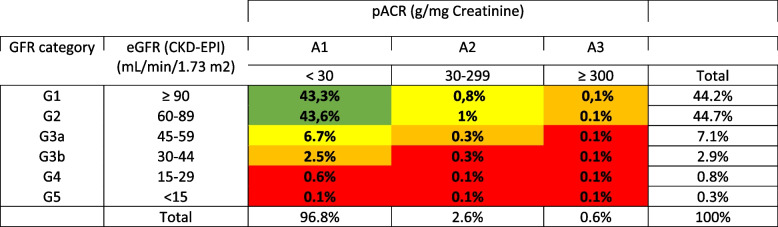
Distribution of CKD Risk by KDIGO Stratification, *N*=2,660,805

**Fig. 2:**
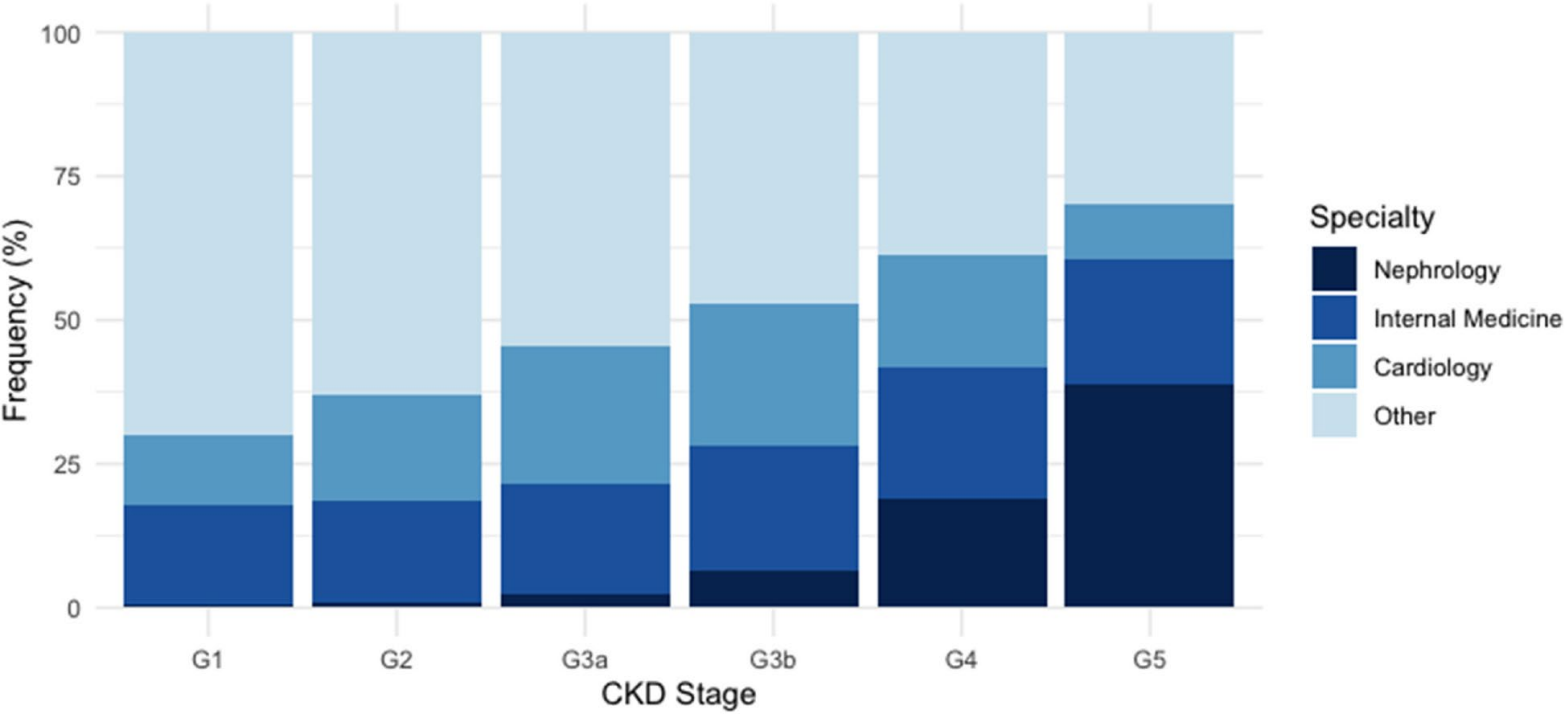
Distribution of Medical Specialties Ordering Serum Creatinine Tests by CKD Stage

The medical specialty that requested most of the creatinine tests was Internal Medicine (17.7%), followed by Cardiology (16.05), Obstetrics/Gynecology (OBGYN) (16.0%), and Endocrinology (8.8%); Nephrology contributed with only 1.1% of the test requests (Fig. 2). OBGYN (20.7 and 13.0%), Internal Medicine (17.2 and 17.7%), and Cardiology (12.3 and 18.6%) ordered most of the creatinine tests with results suggesting no mild CKD (G1 and G2), respectively. At G3a, Cardiology (23.2%), Internal Medicine (18.8%), and Endocrinology (8.7%) led test orders, whereas at G3b, Nephrologists (12.5%) surpassed Endocrinologists in the third position (Fig. 2). Even at G4, Internal Medicine (14.9%) and Cardiology (13%) ordered more creatinine tests than Nephrology (third, 12.5%). Finally, at G5, most tests were requested by Nephrology (39%), followed by Internal Medicine (21.7%) and Cardiology (9.4%). pACR was more often assessed by Internal Medicine specialists (18.1%), Cardiologists (16.2%), OBGYN (9.9%), and Nephrologists (5.9%).

**Fig. 3:**
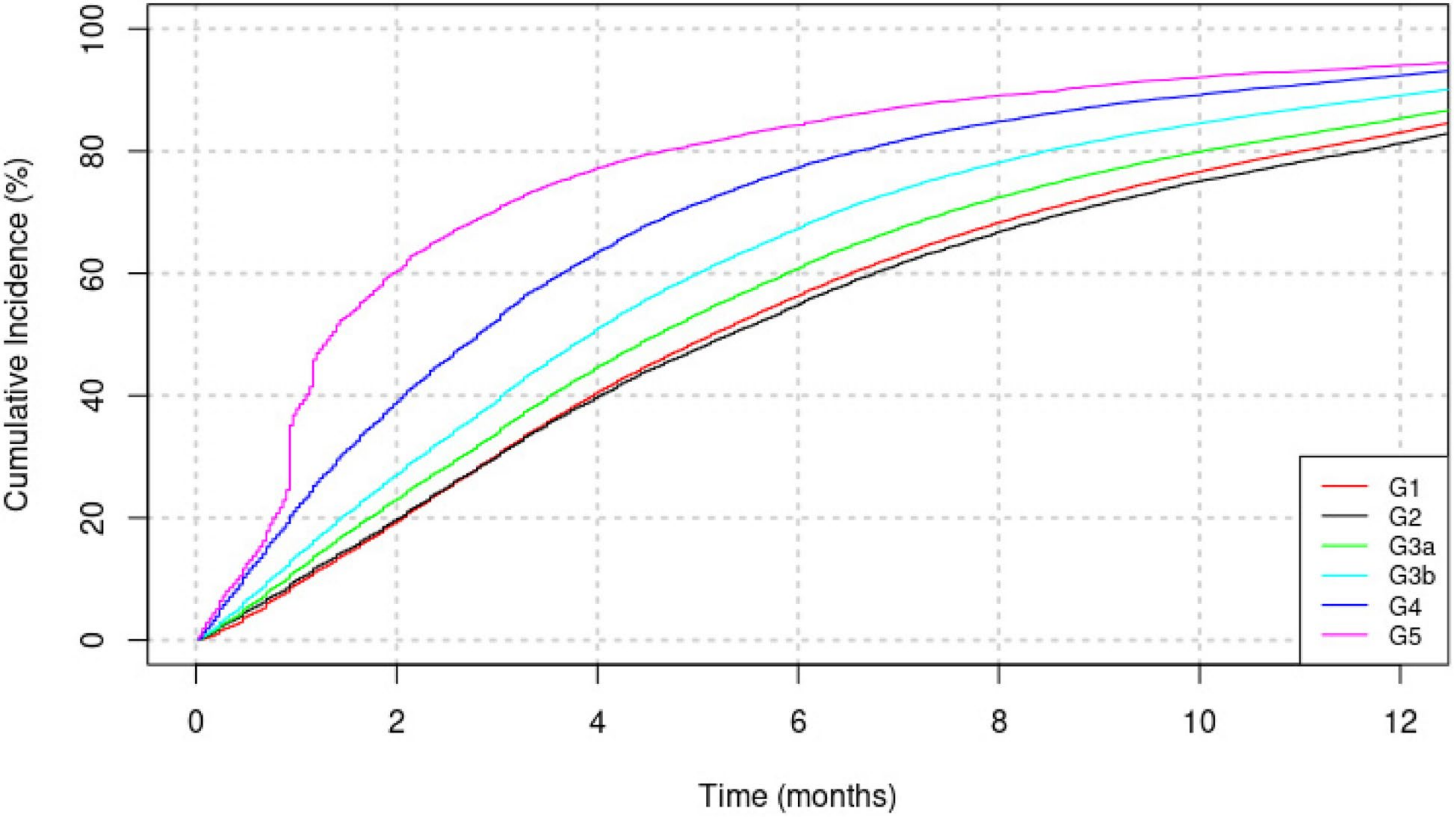
Cumulative Incidence of a New Measurement of Creatinine by CKD Stage

The one-year cumulative incidence functions for repeated creatinine measurement among patients with at least two serum creatinine tests in the sample are shown in Fig. 3. Patients with lower eGFR have a greater probability of having a follow-up serum creatinine test. Patients with CKD stage G3b had probability of approximately 60% of being tested with a new creatinine in 6 months. Among patients with G4, this probability was close to 78%, higher than among patients on stages G2 or G1 (57%). CKD stage 5 patients had the highest cumulative probability of having a new eGFR assessment within 6 months after the first creatinine measurement. In general, patients with CKD had a probability between 80% to 96% of having a second creatinine test within 12 months from the first assessment.

## Discussion

In this nationwide real-world study of practice patterns for kidney function assessment in Brazilians covered with medical insurance, we found that 41% of individuals undergoing creatinine testing lack an accompanying urinary test, a combination of tests that would comply with international guidelines. This incomplete CKD assessment is more common in patients with higher eGFR, who are often not followed by kidney specialists, and presumably are at lower risk of CKD. Most laboratory evaluations of kidney function are performed by non-nephrologists, namely internists, cardiologists and OBGYNs, even among patients with presumable advanced CKD. Finally, up to 80% of patients with at least two eGFR assessments have a follow-up creatinine test within 12 months of the first; the lower the eGFR, the more frequent the creatinine assessment.

Although our study was not designed to evaluate the prevalence of CKD, our results suggest that approximately 11% of patients in Brazil have CKD, assuming no false positive results due to single eGFR and pACR assessments. These results are consistent with the estimates of 10-12% worldwide CKD prevalence [9]. They are also similar to two prior investigations of CKD prevalence in Brazil; one estimated an 8% prevalence in a cohort of civil servants [10], and the other reported an 11.4% prevalence in a convenience sample from a working setting [11].

In Brazil, a country with universal public coverage of healthcare services, nearly 150,000 (640 per million population) patients are on kidney replacement therapy (KRT) as of 2020 [12]. The number of patients on KRT in the country have steadily increased by 6% per year over the last two decades [13]. In this period, mortality rates among patients on hemodialysis, the most frequent KRT modality in the country, increased from 17% to 20% [12]. CKD and its associated conditions consume about 13% of all public health resources in Brazil [14].

Our results imply that approximately 21 million persons are living with CKD in Brazil, a country with roughly 4,000 registered nephrologists. Thus, it is expected that non-nephrologists will screen and assist most CKD patients in Brazil, even those with more advanced stages [13],a problem not exclusive to non-high-income countries [15].In fact, our analysis suggests that, in absolute numbers, nephrologists contribute to approximately 1% of eGFR and pACR assessments in the private setting in Brazil. In general, OBGYN physicians ordered a large absolute number of eGFRs and pACRs, which may be explained by the fact that OBGYNs lead an important fraction of primary care among women in Brazil. In absolute numbers, nephrologists ordered most eGFR assessments only among patients with single eGFR ≤ 15 mL/min/1.73 m^2^. In stages G3a and G3b, in which new therapies that prevent kidney failure can be implemented [5, 6], cardiologists and internal medicine specialists were the most frequent physicians to monitor or screen for CKD. The relatively low absolute contribution of nephrology to CKD screening or monitoring may be explained by the referral structure in Brazil, in which patients with eGFR below 30 mL/min/ 1.73 m^2^ are referred to nephrologists*.* Additionally, our results suggest that several patients with eGFR below 15 mL/min/ 1.73 m^2^, who were mostly monitored by nephrologists, were tested for urinary albuminuria. The value added by albuminuria measurements in this advanced CKD population is uncertain because no interventions to reduce albuminuria have been proven to delay CKD progression in this context [4]. Thus, this practice may not necessarily reflect the most cost-efficient strategy to monitor advanced CKD patients. Our analysis suggests that efforts to increase CKD awareness and promote screening should target non-nephrologists in absolute terms but should also focus on incentivizing best practices among nephrologists. However, there are conflicting recommendations for CKD screening among distinct societies representing medical specialties.

Diverse guidelines from cardiology, internal medicine, and nephrology have shaped the varied implementation of CKD screening, mostly diverging on priorities. The American College of Physicians (ACP) advises against CKD screening in the general population due to a lack of evidence on its risks and benefits, warning it may lead to overdiagnosis and added costs [16]. KDIGO highlights that using cystatin-C to measure eGFR can reduce misclassification and overdiagnosis risks, advocating for CKD screening in patients with a wide array of risk factors for CKD [4]. Emphasizing the clinical advantages of early interventions, KDIGO supports the prioritization of efforts to implement CKD screening [4]. Meanwhile, the 2021 European Society of Cardiology (ESC) Guidelines recognize CKD as a significant cardiovascular risk factor, suggesting its evaluation in broader cardiovascular screening [17]. They underline the value of albuminuria and low GFR results, which can inform strategies, especially with new treatments that reduce cardiovascular events and preserve kidney function [17]. Reflecting the ESC's stance, the European Renal Association now urges a more proactive CKD screening in the wider population [18].

However, barriers to broader CKD screening and monitoring prevail. Our analyses indicate that up to 41% of patients who had an eGFR evaluation did not undergo a reassessment of kidney health within 12 months. This pattern echoes findings from US and UK studies among CKD patients [19, 20]. The proportion of patients undergoing evaluations for eGFR and pACR grows with the severity of CKD stages, likely due to the increasing involvement of nephrologists in advanced CKD care. In fact, a US survey found that non-nephrologists often underestimate the significance of albuminuria in early CKD stages [21]. This uncertainty can hinder timely education, planning, and interventions critical for CKD progression and cardiovascular risk reduction [4]. Importantly, even among patients with a low risk for kidney failure (e.g., those with eGFR > 60 mL/min/ 1.73 m^2^), many may still have CKD according to KDIGO guidelines [4]. Our results suggest that approximately 54% of patients with eGFR > 60 mL/min/ 1.73 m^2^ were tested with a urine test. Among this tested population with eGFR > 60 mL/min/ 1.73 m^2^, approximately 2.3 % had results consistent with CKD (*N*= 59,818). The absence of an albuminuria measurement in this context could result in missed diagnoses of CKD. 

This study has several noteworthy limitations. First, we relied on secondary data extraction from an administrative database for our study. We did not have detailed clinical information on participating patients, nor were we able to assess the reasons for laboratory evaluation. Therefore, we were unable to evaluate whether creatinine or albuminuria were ordered for screening or monitoring purposes. Second, our evaluation of medical specialties is limited to the reported information on the laboratory requisition. In Brazil, internal medicine may broadly cover unspecialized physicians working on primary care, specialists in primary care (family medicine), or specialists in internal medicine. Third, our database covers primarily insured patients, not capturing users of the unified public healthcare system in Brazil. This can explain the lack of family medicine practitioners as a common medical specialty requesting laboratory tests in our sample, as these physicians are more often leading the care for patients in the public healthcare system. Fourth, the patient population in this study represents relatively young and healthy patients, who are presumably under a low risk for CKD. Therefore, our results do not necessarily generalize to higher risk settings in Brazil. Fifth, our sample mainly comprised patients from the Southeast region in Brazil, which may limit the external validity of our findings. Sixth, the GFR was estimated by the CKD-EPI equation although serum creatinine was measured by the Jaffé method instead of the isotope dilution mass spectrometry (IDMS)-traceable Jaffé method. Finally, we used single assessments of creatinine and pACR to define CKD. Despite these limitations, our results have key strengths. Our large sample covers a wide geographic distribution in Brazil, which increases the external validity of our findings. Additionally, our results are highly consistent with prior studies, which further reinforces the advantages of using large real-world databases for epidemiological research. Therefore, we believe our data can support action plans to reduce the growing burden of CKD and its complications in Brazil.

In conclusion, in this real-world study evaluating practice patterns for laboratory evaluation of biomarkers of kidney damage and function, we found that non-nephrologists are the key drivers of CKD assessment in Brazil and that CKD assessment is largely non-compliant to current guideline recommendations, since more than one-third of patients with eGFR evaluation lack urinary tests. Our results suggest that a large proportion of patients with CKD remain undiagnosed in Brazil, particularly those with proteinuric CKD with eGFR above 60 mL/min/1.73 m2. Furthermore, most patients at risk of progressing to kidney failure and suffering poor outcomes are sub-optimally screened and risk-stratified in Brazil. Increasing the awareness of the importance of CKD diagnosis and coordinating multidisciplinary efforts for screening, risk stratification and referral strategies remain unmet goals in CKD care that require urgent attention.

### Supplementary Information

**Supplementary Material 1.**

## Data Availability

The datasets used and/or analyzed during the current study are available from the corresponding author on reasonable request.
