# Supplementary figures and images for: Patterns of kidney function and risk assessment in a nationwide laboratory database: the Brazilian CHECK-CKD study
Source: BMC Nephrol. 2024 Jun 4;25:191. doi: 10.1186/s12882-024-03588-w (PMC11149244; doi:10.1186/s12882-024-03588-w)

Supplementary Material

**Supplementary Figure 1.** Distribution of Observations by Region


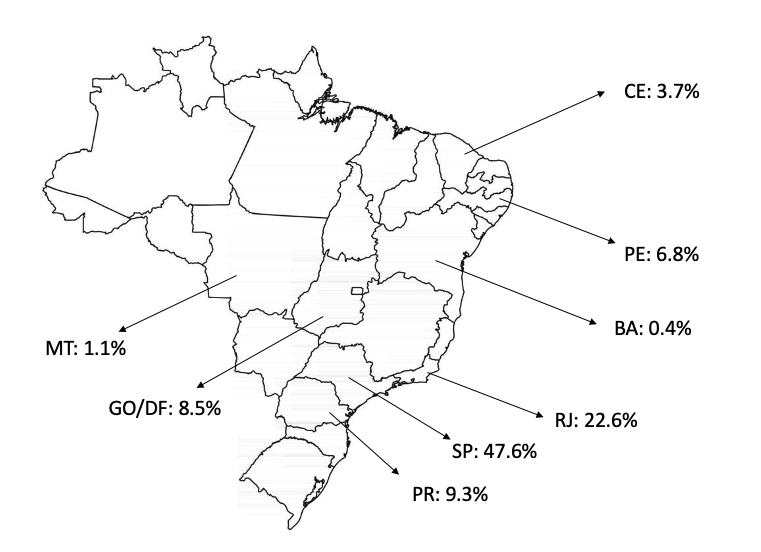

Supplement: Supplementary file 1 — Supplementary Material 1. [file 12882_2024_3588_MOESM1_ESM.docx]
